# Cane Toads on Cowpats: Commercial Livestock Production Facilitates Toad Invasion in Tropical Australia

**DOI:** 10.1371/journal.pone.0049351

**Published:** 2012-11-07

**Authors:** Edna González-Bernal, Matthew Greenlees, Gregory P. Brown, Richard Shine

**Affiliations:** School of Biological Sciences A08, University of Sydney, New South Wales, Australia; Helmholtz Centre for Environmental Research - UFZ, Germany

## Abstract

Habitat disturbance and the spread of invasive organisms are major threats to biodiversity, but the interactions between these two factors remain poorly understood in many systems. Grazing activities may facilitate the spread of invasive cane toads (*Rhinella marina*) through tropical Australia by providing year-round access to otherwise-seasonal resources. We quantified the cane toad’s use of cowpats (feces piles) in the field, and conducted experimental trials to assess the potential role of cowpats as sources of prey, water, and warmth for toads. Our field surveys show that cane toads are found on or near cowpats more often than expected by chance. Field-enclosure experiments show that cowpats facilitate toad feeding by providing access to dung beetles. Cowpats also offer moist surfaces that can reduce dehydration rates of toads and are warmer than other nearby substrates. Livestock grazing is the primary form of land use over vast areas of Australia, and pastoral activities may have contributed substantially to the cane toad’s successful invasion of that continent.

## Introduction

Human activities and habitat perturbation often facilitate the success of invasive species, either because invasive species are better able to tolerate the changes that have been wrought [1], or because those anthropogenically-driven changes provide resources that are useful to the invader [2], [3]. For example, agricultural practices such as soil disturbance and the addition of fertilizers often facilitate the establishment of non-native plants [4]. More generally, a high proportion of the most successful invasive species worldwide are commensal taxa: those that live in close association with humans, and thrive under conditions unsuitable for most native taxa [5]–[7].

Although commensal taxa that exploit even densely-populated cities (such as several rodent taxa) are the most obvious examples of invaders benefiting from human-wrought modifications to habitat, cities occupy only a small proportion of the land surface in most parts of the world. Agricultural activities alter much larger areas of land. In terms of spatial scale, livestock production stands out as the major issue in many countries. For example, more than half of the Australian continent is used for grazing livestock (around 4,301,008,000 ha [8]–[10]). Hence, any ecological impacts of grazing activities are of great interest, especially in a continent like Australia where invasive species have exerted major impacts on the native biota [4], [11]–[14]. The role of livestock grazing in promoting plant invasions has been well studied [15]–[17], but much less is known about the effects of livestock grazing on the spread of invasive animals.

Our study focuses on a large anuran (originally from South and Central America) that was introduced to Australia in 1935, and has since spread across much of the continent, with severe ecological impacts on native fauna [14], [18]. The spread of cane toads has been facilitated by habitat changes wrought by the livestock-grazing industry, notably the provision of additional water sources [19], [20] and of open linear corridors that facilitate rapid dispersal [2]. In the current paper, we examine an additional habitat modification due to livestock production: the cattle feces (“cowpats”) that are liberally scattered across the landscape. Serendipitous observations at our study site in tropical Australia showed that toads often aggregate on and near cowpats, suggesting that the invaders somehow benefit from the presence of those fecal piles. We conducted research to quantify toad usage of cowpats, and to test alternative hypotheses about the nature of any benefits that cowpats might confer to toads.

## Materials and Methods

### Ethics Statement

All procedures were approved by the University of Sydney Animal Care and Ethics Committee (Protocol # L04/4-2009/3/4999).

### Study Species and Site

Cane toads (*Rhinella marina*; formerly *Bufo marinus* Linnaeus 1758) are large (at our study site, adults average 11 cm snout-urostyle length [SUL], 150 g) toxic bufonid anurans. Following their introduction to northeastern Australia in 1935 in a futile attempt to control insect pests of sugarcane plantations, the toads have spread southwards and westwards across about one-quarter of the Australian continent [21], [22]. Their westward expansion has taken them into regions that are hotter and seasonally much drier than those within the species’ native range [23], [24]. The toads deal with those dry-season challenges by modifying their activity levels [23], and utilizing anthropogenically-provided sources of water to maintain hydration [19]; the water permeability of their skin also has shifted in ways that facilitate persistence in arid environments [24].

We studied cane toads on the floodplain of the Adelaide River, 60 km east of the city of Darwin in the Australian wet-dry tropics (12°38’S, 131°19’E). The area experiences high temperatures year-round (mean daily maxima >30°C in all months), but with precipitation concentrated in a four-month wet-season (December to March). Previous papers provide detailed information on landforms, climate and toad biology at this site [19], [25]–[28]. Most of our work was carried out on Beatrice Hill Farm, a property used for grazing of Brahman cattle and domesticated water buffaloes. The farm consists of managed paddocks of pasture grasses on a relatively open floodplain, with patches of eucalyptus and monsoon forest along the floodplain fringes. Cane toads are common throughout the landscape, having reached the site in 2005 (6 years before our study).

### Field Surveys of Cane Toad Distribution Relative to Cowpats

During the dry season (September 2010), we surveyed 11 transects, each measuring 100 m long and 2 m wide, all in open pastures, over a three-night period (2000–2200 h each night). The transects were located at three different areas that were separated by a distance of 250 m. The areas were selected based on the presence of cattle for at least 3 weeks, which would allow the presence of cowpats. The first area contained 3 transects, the second 4 transects and the third 4 transects. All transects were parallel and they were separated by a distance of 15 m. For each toad located inside each transect, we recorded body temperature, posture, and distance to the nearest cowpat within the transect area. The numbers and linear dimensions of cowpats were also recorded. To measure the thermal environment available to toads, we recorded the surface temperature of each cowpat and the adjacent ground temperature 1 m away using an infra-red thermometer (Dick Smith Model Q1370).

To determine if the toads were closer to cowpats than expected by chance (given the relative numbers of toads and cowpats on each transect), we performed a randomization test (details below). Based on 5000 randomized replicates, we calculated the frequency distributions of expected distances from each randomly generated toad location to the nearest randomly generated cowpat location. We then compared that expected distribution to the distances actually recorded.

### The Effects of Cowpats on Toad Hydric Balance

Plausibly, cowpats may provide a moister microhabitat than the surrounding soil, thus enabling toads to retain or gain water. To test this hypothesis, we constructed models of toads from 2% agar; such models accurately predict rates of water loss and gain of live toads, while removing the confounding effects of toad posture and behavior on these variables [29]. We used small agar models to represent juvenile cane toads, and larger models to represent adult toads (3.2×3.9×3 cm and 6.4×7.8×3 cm respectively). We deployed the models outdoors in a pasture area (2 km from the sites where we surveyed toads) on seven different substrates in a 7×7 latin square design (each substrate type equally represented in all rows and all columns). Four of the substrate types were cowpats, of varying ages since deposition (0, 24, 48, 72 h); the other substrates were bare soil, elevated mounds of bare soil, and grass. To create cowpats, we collected freshly-deposited buffalo feces from handling yards at the farm, and kept the material in closed buckets. The 72-hour-old cowpats were deployed three days before we commenced trials with the agar models; the 48-hour-old cowpats were deployed the next day, and so forth. Our artificial cowpats were all approximately 32×21 cm, and 7 cm high, based on the mean values of 50 natural cowpats that we measured in the field. Because post-metamorphic cane toads are active nocturnally [30], [31] we deployed the agar models overnight, from 1830–0630 h. Models were weighed at the beginning and the end of this 12-hour period to measure rates of water loss or uptake (expressed as a proportion of initial mass). We ran three sets of trials, measuring a total of 49 agar models of each size class per trial. We used ANOVA to evaluate the effects of substrate types and model sizes on the extent to which the agar models changed in mass over the course of their deployment.

### The Effects of Cowpats on Toad Feeding Rates

Plausibly, access to a cowpat might enhance a toad’s feeding opportunities (e.g. if edible insects are attracted to bovine feces). To test this hypothesis, we constructed 12 metal-sided outdoor enclosures (each 2.4×1.2 m, with 1-m high walls) with a substrate of natural soil and vegetation. We collected 36 toads from the Adelaide River floodplain and kept them without food for 48 hours (to empty their digestive tracts) prior to the experiment. Each toad was then placed individually at dusk into an outdoor enclosure that either had a cowpat (constructed using the same methods and same dimensions as above), a mound of bare soil the same size and shape as a cowpat, or had neither a cowpat nor a soil mound. Treatments were randomized within the array of enclosures. The next morning, we collected and humanely euthanized the toads via an overdose of pentabarbitol administered by intracoelomic injection, and dissected them to record the numbers and types of prey in their stomachs. These data were analyzed with an ANOVA, with treatment (enclosure type) as the factor.

## Results

### Field Surveys of Cane Toad Distribution Relative to Cowpats

Overall, we recorded a total of 26 toads and 177 cowpats in the 11,200×m^2^ survey area. Based on the mean size of a cowpat, an average of 0.41% of the ground was covered by cowpats. Despite this low availability, 17 of the 26 toads were sitting on cowpats when we found them; thus, toads were found on cowpats more often than would be expected by chance (χ^2^ = 2595.27, p<0.0001). In 5 of the 11 transects, we observed a total of 8 toads that were not sitting on cowpats (3 in one transect containing 23 cowpats, 2 toads in one transect containing 21 cowpats, one toad in a transect containing 9 cowpats, one toad in a transect containing 21 cowpats and one toad in a transect containing 8 cowpats), and we measured the distance from each of the 8 toads to the nearest cowpat. We conducted a randomization test by randomly generating 5000 placements of toads and cowpats in the observed combinations (3 toads with 23 cowpats, 2 toads with 21 cowpats,… etc) and measuring the distance between each randomly generated toads and the nearest randomly generated cowpat (we included a 30 cm buffer around each random cowpat ‘point’ to approximate the diameter of a cowpat).

On average, these toads were 97.5±59 cm (mean ± SE) from the nearest cowpat that was in the survey area (range 5–500 cm). This observed value ranked as the 46th lowest value when ranked among the 5,000 randomly generated minimum distances (mean 300±1.75 cm) Based on these simulations, the observed result was extremely unlikely to have occurred by chance (p<0.009). Thus, toads were found on cowpats more often than expected by chance, and toads that were not on cowpats were closer to them than would be expected by chance.

### Body Temperatures of Toads on Cowpats

**Figure 1:**
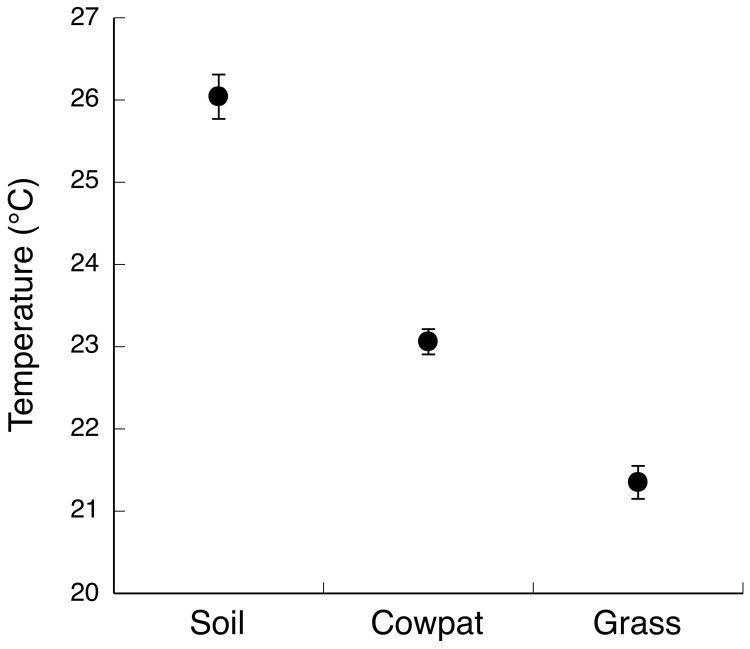
Differences in surface temperatures of three different substrates available to cane toads at our study site. The graph shows mean values and associated standard errors.

The mean body temperature of the toads on cowpats (21.76±0.4°C) was almost identical to that of toads that were not on cowpats (21.74±0.54°C; F_1,24_ = 0.0009, p = 0.98), and the mean temperatures of cowpat surfaces were similar to those of adjacent ground surfaces (22.77±0.37°C vs. 22.53±0.37°C respectively; F_1,50_ = 0.20, p = 0.66). At a broader spatial scale (at the level of the entire transect), however, our analyses revealed substrate-dependent variation in mean temperatures, with cowpats intermediate in this respect between soil substrates (the warmest) and grass substrates (the coolest; F_2,332_ = 97.55, p = 0.0001; Figure 1).

### The Effects of Cowpats on Toad Hydric Balance

**Figure 2:**
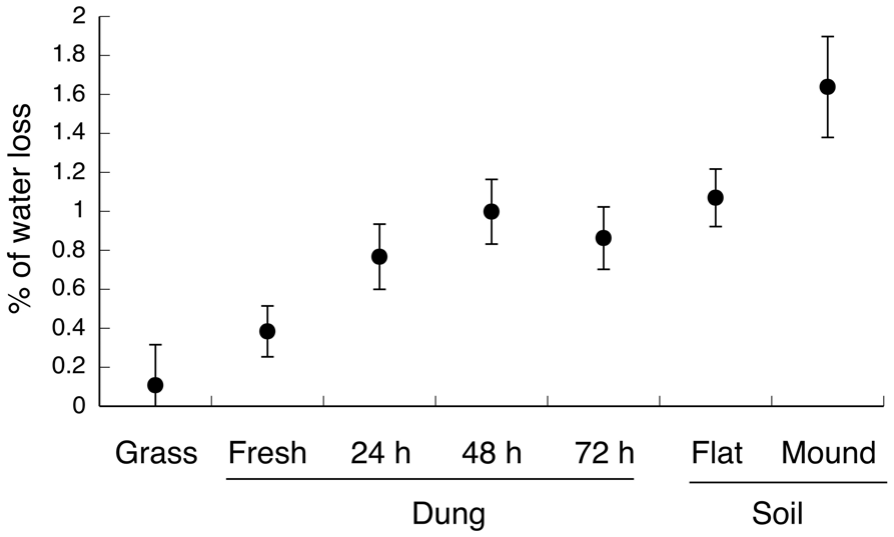
Influence of substrate type on the percentage of water loss of agar models during overnight observations (over a 12-hour period). The graph shows mean values and associated standard errors.

Most agar models showed only minor changes in mass over the 12-hour experimental period (0.75±0.8% mass loss, range −3.7–1.8%), with most models losing rather than gaining water. Live grass provided the best buffer to moisture loss, followed by fresh dung. Models on older cowpats lost more water, and models on soil desiccated even faster (F_6,278_ = 7.30, p<0.0001; Figure 2).

### The Effects of Cowpats on Toad Feeding Rates

**Figure 3:**
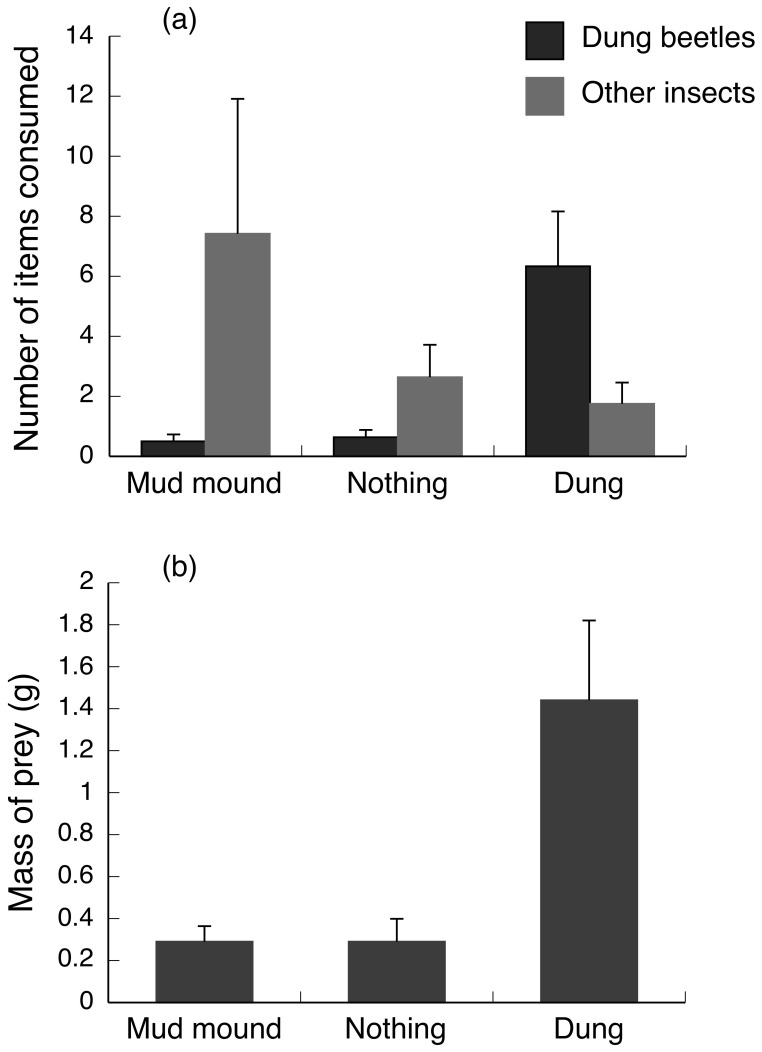
Influence of access to cow dung on the feeding rates and dietary composition of cane toads in outdoor enclosures. (a) Total number of prey items consumed per toad, divided into dung beetles and other insects. (b) Average mass of prey consumed per toad. The graphs show mean values and associated standard errors.

In outdoor enclosures, the presence of an artificial cowpat increased cane toad feeding success. Cowpat presence resulted in toads consuming more dung beetles (F_2,34_ = 9.25, p = 0.0007; see Figure 3) and a greater overall mass of prey (F_2,34_ = 8.01, p = 0.002; Figure 3). Excluding dung beetles, the number of insects consumed by toads (mostly ants) did not differ significantly among treatments (F_2,34_ = 1.24, p = 0.30).

## Discussion

Not only do habitat change and invasive species both contribute to the loss of biodiversity [32], but the two processes also interact in important ways [33]. Our results support the idea that livestock grazing – the most widespread form of land-use in Australia – has facilitated the success of a toxic invasive anuran. Not only have the water sources and dispersal pathways provided by farms aided cane toads in their spread across the Australian tropics [2], [20]; cattle and water buffalo production also inevitably results in the accumulation of cowpats, and cane toads derive multiple benefits from that material.

Much of the territory colonized by cane toads in Australia, including our own study area, experiences more prolonged and intense dry periods than occur in the natural distribution of cane toads [19]. The long dry-season means that toads must have access to water to maintain their hydration state, and the abundance of food (insects) also falls dramatically at this time of year [19] at the same time as nocturnal minimum temperatures decrease to levels that constrain toad activity [34]. Even in well-watered agricultural areas, available sources of surface water may be separated by long distances, conferring substantial risk for an anuran that moves away from water (perhaps because that water source disappears due to prolonged dry conditions [28]. Under these conditions, the dung deposited by cattle and buffalo may provide critical resources that sustain toad hydric and nutritional balance, essentially providing connections across the arid regions that separate patches of favorable (well-watered) habitat. Similarly, even if the only available water is in elevated stock-troughs inaccessible to cane toads (as occurs over wide areas), bovine defecation essentially transfers that water to ground level, and thus provides toads with a microhabitat where they can replenish their body moisture.

Cowpats provide a nutritional as well as hydric benefit. In our enclosures, the dung beetles attracted to cowpats provided an abundant food source for toads. Our work was conducted in the dry season, when cane toads exhibit reduced rates of feeding and growth because of the scarcity of edible insects [19]. Under these circumstances, cowpats may offer more favorable foraging microhabitats than any adjacent sites. It is difficult to evaluate the thermal consequences of cowpat use, but we note that a toad on bare ground (the most widespread substrate in our study area) would experience low body temperatures as well as high rates of water loss.

Although some of our experiments relied upon enclosures, and others used agar models instead of live toads, the results are likely to be relevant to free-ranging animals. Previous research has shown that agar models provide robust estimates of water exchange rates in free-living cane toads in our study areas [27], [35], [36]. Our enclosures were situated within an area containing abundant free-living toads (and farms, and thus manure), and it is difficult to see why the facilitation of food supply offered by our artificial cowpats would not occur in nature also. Cane toads have been observed consuming dung beetles in nature [37], and dissections of field-collected toads often have revealed dung beetles among the stomach contents [38].

Cattle production can negatively affect native ecosystems in several ways: for example, by degrading habitat quality, reducing the abundance of native shrubs, changing vegetation composition and structure, and promoting weed invasion [9], [39], [40]. Our study identifies another way in which cattle raising facilitates the persistence of an invasive vertebrate, the cane toad, by providing resources that would otherwise be scarce during the prolonged dry-season. Given that a cow can produce 12 cowpats per day [37], a simple calculation based on cattle abundance (28.5 million cattle in Australia in 2006 [8]) suggests that over 300 million cowpats per day are deposited on Australian soil. The degree to which those cowpats affect toad populations remains unknown, and doubtless varies among regions, and depends upon weather conditions. Because the majority of areas devoted to commercial cattle production lie within relatively arid regions of Australia, we suspect that cattle feces may be a more important resource in at least some of those areas than is the case in the wet-dry tropics. Cowpats may be less significant to toads in well-watered, less seasonal regions; but cattle production is rarely a major industry in such places [41].

The wet-dry tropics also contain a diverse array of native anurans, but we have never seen them sitting on and near cowpats in the way that we have so often observed in the invasive toads. Part of the reason for that disparity may lie in the abundance of cane toads in disturbed habitats, which often contain few native anurans [18], [42]. Also, most of the native anurans that inhabit disturbed areas (buildings, etc.) in the wet-dry tropics are treefrogs (e.g. *Litoria caerulea, L. rothii, L. rubella*) that spend relatively little time on the ground; and hence, are in less direct contact with cowpats than are the (entirely terrestrial) cane toads. Like many invasive organisms, the cane toad exhibits highly flexible behavior, and an ability to exploit novel resources within its invaded range [43]. The use of cowpats represents just such a flexibility; although the native range of cane toads contains large mammals that deposit large fecal piles (unlike Australian mammals [37], [44]), we doubt that cowpats (or their equivalents) have played an important role in cane toad biology over evolutionary time.

Understanding the proximate mechanisms by which habitat disturbance facilitates biological invasions remains a major challenge for conservation biologists [1]. In the system that we have studied, the interactions are complex, and mediated by human interference at several levels. The primary taxa involved in those interactions – cattle, dung beetles and cane toads – are all introduced species, brought intentionally to Australia for reasons closely linked to agricultural industries [9], [18], [37]. In the case of both dung beetles and toads, the success of their invasion has certainly (beetles) or plausibly (toads) been enhanced by the prior introduction of a distantly related taxon. Indeed, any enhancement of the cane toad’s success in Australia by virtue of its ability to exploit the resources offered by cowpats, is clearly dependent upon both of those previous introductions (i.e. of both cattle and dung beetles). More generally, the “invasional meltdown” hypothesis suggests that changes wrought by earlier invasions can facilitate establishment of later-arriving species [45] (but see [46], [47] for critiques of evidence for this phenomenon). The contrary phenomenon can occur also, whereby an earlier invasion induces biotic resistance against a later-arriving invader, thus restricting its spread [48]; or an earlier invasion preadapts the native biota in ways that reduce the later invader’s ecological impact [49]. In the case of cane toads within Australia, a broad range of anthropogenic modifications have contributed to this species’ success even in formidably arid climates. The ability to utilize the nutritional, hydric and thermal opportunities offered by cattle feces may well have been a significant element of that success.
